# Compliance with the 24-hour movement guidelines and weight status: results from 40,970 adolescents

**DOI:** 10.3389/fpubh.2024.1472188

**Published:** 2025-03-07

**Authors:** Yang Su

**Affiliations:** School of Humanity and Physical Education, Nanjing Sport Institute, Nanjing, China

**Keywords:** 24-hour movement, 24-hour movement guidelines, weight status, Youth Risk Behaviour Survey, adolescent

## Abstract

**Background:**

Childhood obesity, which has been associated to heart disease, metabolic syndrome and disorders such as feelings of worry and sadness in children is one of the prominent obstacles for the health of the general population in the recent decades. A great deal of research shown the connection between meeting the 24-hour movement guidelines and weight status in young people. The purpose of this study is to find the correlation between compliance with the 24-hour movement guidelines and weight status in a large collection of U.S. teenagers, and to examine whether these connections vary by sex, age, or ethnicity/race.

**Methods:**

The study was gathered from the Youth Risk Behaviour Survey (YRBS) conducted in 2017, 2019, and 2021, the final analysis used a total of 40,970 participants aged 14–17 years. The study used logistic regression analysis to estimate the correlation between adherent to the 24-hour movement guidelines (independent) and weight status (dependent) while adjusting for sex, age, race/ethnicity, grade, eating habits, cigarette use, alcohol drinking, perception of weight, weight loss, sports team participation, and year of data acquisition. For statistical significance, a *p*-value <0.05was used.

**Results:**

Participators who not meeting any guidelines (OR = 1.38, CI = 1.20–1.58, *p* < 0.001), 1 guideline (OR = 1.42, CI = 1.28–1.58, *p* < 0.001), and 2 guidelines (OR = 1.18, CI = 1.20–1.58, *p* < 0.001) were more associated with worse weight status, compared with those who met the 3 guideline. For boys, who did not meet any of the guidelines (OR = 1.63, CI = 1.37–1.93, *p* < 0.001), 1 guideline (OR = 1.49, CI = 1.31–1.70, *p* < 0.001), and 2 guidelines (OR = 1.16, CI = 1.00–1.34, *p* = 0.048) were correlated with unfavourable weight status compared with who met all guidelines were more associated. The disaggregated results for gender, age, race, and ethnic group shows that the impact of not adhering to movement guidelines is more pronounced in boys than in girls, each age group demonstrates a trend where not meeting guidelines correlates with poorer weight status, White and Hispanic/Latino participants exhibit stronger negative outcomes from poor guideline adherence compared to other groups.

**Conclusion:**

This research suggests that meeting the 24-hour movement guidelines can significantly aid in averting weight-related problems among U.S. adolescents, with pronounced differences across sex, age, race/ethnicity subgroups. To validate these preliminary findings, future research should employ longitudinal designs to examine the differences among various age groups, sexes, and races, and to determine if promoting adherence to these movement guidelines effectively mitigates weight-related issues during adolescence.

## 1 Introduction

Over the last several years, the frequency of obesity and overweight among preschoolers, children and teenagers had a strong uptrend all over the world. One of the most prominent difficulties for public health in the recent decades is Childhood obesity because of its long-term negative effects (1). In 2016, there were over 340 million teenagers and children aged 5 to 19 who were diagnosed with obesity around the world (2). The prevalence of obesity increases by a factor of 1.5 between 2012 and 2023 compared to 2000 to 2011 (3). The annual medical expenses associated with childhood obesity are notably high, with costs for obesity-related care averaging $307.72 and those for overweight care averaging $190.51, compared to their healthy-weight counterparts (4). Obesity in children is associated with an increased likelihood of early puberty, irregular menstrual period in adolescent girls, sleep apnoea that obstructs breathing and various cardiovascular risk factors such as prediabetes, type 2 diabetes, elevated cholesterol levels, high blood pressure, non-alcoholic fatty liver disease, and metabolic syndrome (5). Additionally, children and adolescents who are overweight have higher risk of psychological problems, for example, anxiety disorder, depression, poor disturbance of body image, difficulties in peer relationships, and cynophobia (1). Young individuals who are overweight approximately 5 times more likely to develop obesity adults than their non-obese peers. The possibility of children and adolescents with obesity who grow into adults with obesity is five times as much as their peers who do not have obesity issues. Children and teenagers with obesity also increase the likelihood of morbidity and mortality when they grow up, elevating the likelihood of the occurrence of chronic diseases like type 2 diabetes and heart diseases (1).

Physical activities, sufficient sleep, and reduced sedentary behaviours, especially screen times (ST) have several well-established positive results (6). Toddlers, Kids, teenagers, and young people have the ability to reach various reference guide for exercise, sitting time, and amount of sleep. Canada introduced the 24-hour movement guidelines for individuals between the ages of 5 and 17 years old in 2016, followed by guidelines for the children aged 0–4 years in 2017. According to these guidelines, Children aged one to five should participate in physical activity (PA) for a minimum of 3 hours each day (with 1 hour and above of moderate to vigorous intensity for preschoolers), reduce the amount of time spent on screens to a maximum of 1 hour per day, and between 10 and 13 h of sleep (7). Young individuals should participate in PA that is of moderate to high intensity for 1 hour and above, reduce recreational time spent on screens to 2 hours at most, and should sleep at least 9–11 h o (for those between the ages of 5–13 s) or sleep at least 8–10 h (for those between the ages of 14–17) (8, 9). These comprehensive guidelines enhance previous recommendations by integrating PA, ST, and sleep into one single framework. Their rapid acceptance by the scientific and professional community has led to adoption in numerous nations, such as South Africa, New Zealand, Australia, including, and the Pacific region (10). The 24-hour movement guidelines aim to help young individuals balance daily activities and prevent non-communicable diseases like obesity (11).

A great deal of research shown the connection between meeting the 24-hour movement guidelines and weight status in adolescents. Systematic reviews and meta-analyses have investigated the connection between meeting the 24-hour movement guidelines and signs of obesity in young children, kids, and teenagers (6). Generally, these reviews found that adherence to all three movement suggestions was cross-sectionally correlated with lower entirety indicators related to obesity, indicating better weight status among those who met the guidelines. Specifically, meeting the suggestions was linked to lower odds of obesity/overweight and obesity alone, and lower BMI, zBMI, waistline, and BFP (10, 12). For instance, research conducted by Feng et al. investigated that adherent to the combined 24-hour movement guidelines significantly reduced BMI z-scores and obesity odds (13). Zhu et al. showed that only adhering to the recommended level of PA, or in combination with the other two guidelines, was correlated with the smallest likelihood of being overweight or obese (7). This effect was particularly notable in females, who had a 71% lower chance of being overweight when complying with the PA recommendation alone compared to all three guidelines. A prospective study revealed that lower adherence in childhood was associated with increased levels of body fat during early adolescence and teenage years. For instance, children who did not fulfill any of the criteria had a 1.66 standard deviation higher BMI z-score in early adolescence compared to those who met all components, according to Chemtob et al. (14). These studies concluded that adherent to the integrated 24-hour movement guidelines is crucial for keeping a healthy body weight among youngsters. This underscores the importance of addressing PA, ST, and sleep duration collectively in public health initiatives aimed at combating childhood and adolescent obesity (15). The need for an integrated approach to behavioural recommendations to maximize health outcomes in the young population was emphasized by the research results.

The Youth Risk Behaviour Survey (YRBS) was developed in 1989 to monitor critical health risk behaviours contributing significantly to death, disability, and social issues among U.S. high school students. Conducted by the Centres for Disease Control and Prevention (CDC), this national survey collects data representative of 9th to 12th grade students in public and private schools nationwide (16). Previous studies in this field have predominantly investigated the association between individual behaviours and weight status using YRBS data, particularly focusing on PA and sedentary behaviours (17–20). For instance, Laurson et al.’s study examined PA, sedentary behaviours, sleep, and weight status separately, highlighting the need for future research to integrate these factors comprehensively (18). However, these studies often used data limited to single-year snapshots from a decade ago, potentially overlooking how these factors simultaneously influence obesity and each other over time. The research underscores the necessity for an integrated approach to behavioural recommendations to maximize health outcomes among young people. While these studies contribute significantly to understanding adolescent weight status development and inform preventive strategies comprehensively, they frequently lack nationally representative samples, thereby limiting the generalizability of their findings. Moreover, many studies neglect to account for dietary factors that crucially impact adolescent weight status, potentially compromising the reliability of their conclusions. Based on existing research evidence, this study hypothesizes two points: firstly, that adherence to the 24-hour movement guidelines is negatively correlated with overweight/obesity (OW/OB) in American adolescents, where higher adherence corresponds to a reduced risk of OW/OB. Secondly, it posits that the relationship between adherence to these guidelines and OW/OB varies among American adolescents based on age, sex, and ethnicity/race, revealing distinct patterns across these demographic factors. Thus, the study aims to address these gaps and hypotheses by (1) examining the correlation between adherence to the 24-hour movement guidelines and weight status in a large cohort of American adolescents and (2) investigating whether these associations vary by age, sex, or ethnicity/race. By doing so, it tackles critical gaps in the current literature and informs tailored public health interventions aimed at combating childhood and adolescent obesity.

## 2 Methods

### 2.1 Study design and population

A three-stage cluster sampling design was used to produce a nationally representative sample of students in grades 9–12 who attend public and private schools (21–23). The information was gathered from six iterations of the YRBS conducted in 2017, 2019, and 2021, comprising a total of 45,674 participants. The final analysis used a total of 40,970 participants aged 14–17 years. YRBS surveys high school students across the United States to collect data on various risk behaviours. Students completed a self-administered, paper-and-pencil questionnaire with over 90 questions covering topics such as sex, grade level, race/ethnicity, and misuse of opioids (23). The overall response rate was more than 60% in each round of the YRBS administration. Survey results were adjusted to accurately represent the entire nation. The datasets are nationally representative and include variables for sample design and weights, essential for precise statistical estimation. This research obeyed the Declaration of Helsinki guidelines and received clearance from the CDC’s Institutional Review Board (23). Since the data are publicly accessible and anonymized, this study needed no more ethical approval. More details on the YRBS can be found at https://www.cdc.gov/healthyyouth/data/yrbs/index.htm.

### 2.2 Variables and measurements

The YRBS has demonstrated good reliability and validity in assessing health behaviours. Students have been found to consistently report health risk behaviours over time (16, 24).

#### 2.2.1 24-hour movement guidelines (independent variable)

To examine the adherence to the 24-hour movement guidelines, data on PA, ST, and sleep duration were assessed using the following methods. PA was measured by inquiring how many days participants participated in PA for a minimum of 60 min over the past week. This data was then categorized as “7 days” or “fewer than 7 days” based on the guidelines. Participants who reported “7 days” were considered to have met the PA guidelines. For the years 2017 and 2019, ST was assessed by combining responses from two questions about daily hours spent watching TV and using computers or video games outside of schoolwork. The total ST was classified as either exceeding 2 h or 2 h or less. In 2021, ST was measured with a single question encompassing various digital activities, with responses ranging from less than 1 h to 5 or more hours daily. Adherence to the ST guidelines was defined as 2 h or less per day. Sleep duration was evaluated by asking participants about their average nightly sleep, with adherence determined according to age-specific recommendations: 8–10 h for ages 14–17. Adherent to the 24-hour movement guidelines was categorized in two ways: by the number of criteria fulfilled (i.e., 0, 1, 2, or 3) and by specific combinations of guidelines (i.e., none, PA only, ST only, sleep only, PA + ST, PA + sleep, ST + sleep, PA + ST + sleep) (25).

#### 2.2.2 Weight status

In order to determine body mass index (BMI), participants should provide information about their own weight (How much do you weigh without your shoes on?) and height (How tall are you without your shoes on?), more details on the YRBS questionnaires can be found at https://www.cdc.gov/yrbs/questionnaires/index.html. These BMI values were then changed into percent values derived from the Centres for Disease Control and Prevention (CDC) BMI growth charts. The criteria for classifying overweight and obesity were the age- and sex-specific BMI percentiles of ≥85th and ≥ 95th, respectively (26). Therefore, weight status was categorized into non- overweight or- obesity (coded as 1), overweight (coded as 2), and obesity (coded as 3).

#### 2.2.3 Covariates

The questionnaire collected self-disclosed demographic data, including age category (14, 15, 16 and 17 years), sex (boy/girl), and race/ethnicity (White, Black or African American, Hispanic/Latino, or all other races). The following eating habits were also assessed, including (1) fruit juice drinking, (2) fruit eating, (3) green salad eating, (4) potato eating, (5) carrot eating, (6) other vegetable eating, (7) soda drinking, (8) milking drinking, and (9) breakfast eating. In addition, current cigarette use, current alcohol drinking, and sports team participation were also assessed. Measures and responses of the eating habits variables can be found in additional resources.

### 2.3 Statistical analysis

Prior to formal analysis, to handle missing cases, we selected 20 assumptions made on the foundation of the fundamental principle that the number needs to be the same or greater than the percentage of missing data based on a previous and similar study. Descriptive statistics, frequencies (*n*) and percentages (%), were used to describe the characteristics of the samples. Logistic regression analysis was used to estimate the correlation between adherent to the 24-hour movement guidelines (independent) and weight status (dependent) while adjusting for sex, age, race/ethnicity, grade, eating habits, cigarette use, alcohol drinking, perception of weight, weight loss, sports team participation, and year of data collection. Two sets of models were developed to evaluate the relationships between the quantity of guidelines (“none” as the reference group) and specific combinations of guidelines (“none” as the reference group) with weight status. In addition, moderators were sex (males or females), age (13, 14, 15, and 17 years), race/ethnicity (White, Black or African American, Hispanic/Latino, or all other races), and year of data collection (2017, 2019, or 2021). The national YRBS data uses weighting to adjust for sample size, ensuring that the weighted respondent count matches the unweighted participant count. Statistical analyses were carried out with STATA 18.0 Basic Edition (STATA College, Dallas, Texas, United States). A statistical significance was determined if the two-sided *p*-value <0.05.

## 3 Results

Table 1 shows the unweighted characteristics of the study sample, which includes 40,970 participants. The age distribution shows a higher percentage of 15 and 16-year-olds, making up over 56% of the sample. Grade levels are fairly evenly distributed with a slight decrease in representation by grade 12. The sample is split almost evenly between boys and girls. Racial composition is predominantly White, followed by Hispanic/Latino and Black or African American. In terms of health behaviours, a majority of the sample consumes fruit juice and engages in fruit eating, with lower consumption rates for green salads and carrots. Over two-thirds of participants are classified as non-overweight or non-obese, and lifestyle behaviours such as sports team participation and breakfast eating are common. However, adherence to sleep guidelines and PA guidelines is notably poor. 23.96% of participants were getting 8 h of sleep per day and 24.13% were able to achieve at least 60 min of PA per day. Moreover, 12.35% of participants do not meet any of the criteria, 56.29% met only 1 guideline, 26.48% met 2 guidelines and only 4.88%met three guidelines.

**Table 1:** Sample characteristics of this study (unweighted).

*n* = 40,970	%	95%CI	
Age			
14 years	17.76%	17.39%	18.13%
15 years	28.04%	27.60%	28.47%
16 years	28.29%	27.86%	28.73%
17 years	25.91%	25.49%	26.34%
Grade			
9th	29.38%	28.95%	29.83%
10th	28.91%	28.47%	29.35%
11th	26.08%	25.66%	26.51%
12th	15.63%	15.28%	15.98%
Sex			
Boy	50.54%	50.06%	51.03%
Girl	49.46%	48.97%	49.94%
Race			
White	50.06%	49.58%	50.55%
Black or African American	15.98%	15.63%	16.34%
Hispanic/Latino	21.90%	21.51%	22.31%
All Other Races	12.06%	11.74%	12.37%
Body weight			
Non overweight or obesity	67.75%	67.29%	68.20%
Overweight	16.17%	15.82%	16.53%
Obesity	16.08%	15.73%	16.44%
Fruit juice drinking			
Not drink	30.78%	30.34%	31.23%
Drink	69.22%	68.77%	69.66%
Fruit eating			
Not eat	12.92%	12.60%	13.25%
Eat	87.08%	86.75%	87.40%
Green salad eating			
Not eat	44.64%	44.16%	45.12%
Eat	55.36%	54.88%	55.84%
Potatoes eating			
Not eat	38.69%	38.22%	39.16%
Eat	61.31%	60.84%	61.78%
Carrots eating			
Not eat	56.01%	55.53%	56.49%
Eat	43.99%	43.51%	44.47%
Other vegetables eating			
Not eat	19.88%	19.50%	20.27%
Eat	80.12%	79.73%	80.50%
Soda or pop drinking			
Not drink	29.57%	29.13%	30.01%
Drink	70.43%	69.99%	70.87%
Milk drinking			
Not drink	31.89%	31.44%	32.34%
Drink	68.11%	67.66%	68.56%
Breakfast eating			
Not eat	18.27%	17.90%	18.65%
Eat	81.73%	81.35%	82.10%
Current cigarette use			
Yes	5.36%	5.15%	5.58%
No	94.64%	94.42%	94.85%
Current alcohol drinking			
Yes	25.16%	24.74%	25.58%
No	74.84%	74.42%	75.26%
Feel overweight			
Yes	31.48%	31.04%	31.94%
No	68.52%	68.06%	68.96%
Weight loss			
Yes	46.79%	46.31%	47.27%
No	53.21%	52.73%	53.69%
Sports team participation			
Yes	54.55%	54.07%	55.04%
No	45.45%	44.96%	45.93%
Got 8 or more hours of sleep			
Not meet	76.04%	75.62%	76.45%
Meet	23.96%	23.55%	24.38%
Spent 3 or more hours/day on ST			
Not meet	24.21%	23.80%	24.63%
Meet	75.79%	75.37%	76.20%
At least 60 min/day PA for all 7 days			
Not meet	75.87%	75.45%	76.28%
Meet	24.13%	23.72%	24.55%
Number of guidelines met			
0	12.35%	12.04%	12.67%
1	56.29%	55.81%	56.77%
2	26.48%	26.05%	26.90%
3	4.88%	4.67%	5.09%
Combination of guidelines met			
Meet none	12.35%	12.04%	12.67%
PA only	4.79%	4.59%	5.00%
SB only	46.79%	46.31%	47.27%
Sleep only	4.72%	4.51%	4.93%
PA + SB	12.11%	11.80%	12.43%
PA + Sleep	2.35%	2.21%	2.50%
SB + Sleep	12.02%	11.70%	12.33%
Meet all	4.88%	4.67%	5.09%

PA, physical activity; ST, screen time.

Table 2 displays the weighted sample characteristics, adjusting the distribution to better reflect the population. Similar trends to Table 1 are observed, with minor adjustments in percentages across age, grade, and racial categories. The weighted results also show a consistent distribution in terms of sex, with nearly equal numbers of boys and girls. Health and lifestyle behaviours follow similar patterns, with significant majorities drinking fruit juice, eating fruits, and engaging in other positive dietary habits. Following guidelines for PA and sleep remain low, echoing the challenges highlighted in the unweighted data.

**Table 2:** Sample characteristics of this study (weighted).

	%	95%CI	
Age			
14 years	17.26%	16.19%	18.39%
15 years	27.97%	27.18%	28.77%
16 years	28.14%	27.44%	28.85%
17 years	26.63%	25.82%	27.46%
Grade			
9th	29.61%	28.47%	30.78%
10th	28.37%	27.52%	29.24%
11th	26.14%	25.49%	26.79%
12th	15.88%	14.71%	17.12%
Sex			
Boy	49.92%	48.74%	51.10%
Girl	50.08%	48.90%	51.26%
Race			
White	51.54%	48.18%	54.89%
Black or African American	12.61%	11.19%	14.18%
Hispanic/Latino	24.77%	22.05%	27.70%
All Other Races	11.08%	9.54%	12.83%
Body weight			
Non-overweight or obesity	67.99%	66.87%	69.09%
Overweight	16.09%	15.52%	16.68%
Obesity	15.92%	15.16%	16.71%
Fruit juice drinking			
Not drink	30.90%	29.70%	32.13%
Drink	69.10%	67.87%	70.30%
Fruit eating			
Not eat	12.37%	11.66%	13.12%
Eat	87.63%	86.88%	88.34%
Green salad eating			
Not eat	43.71%	42.37%	45.07%
Eat	56.29%	54.93%	57.63%
Potatoes eating			
Not eat	38.11%	36.95%	39.27%
Eat	61.89%	60.73%	63.05%
Carrots eating			
Not eat	54.73%	53.42%	56.04%
Eat	45.27%	43.96%	46.58%
Other vegetables eating			
Not eat	19.52%	18.65%	20.42%
Eat	80.48%	79.58%	81.35%
Soda or pop drinking			
Not drink	30.06%	28.78%	31.37%
Drink	69.94%	68.63%	71.22%
Milk drinking			
Not drink	31.62%	30.53%	32.73%
Drink	68.38%	67.27%	69.47%
Breakfast eating			
Not eat	17.81%	16.87%	18.79%
Eat	82.19%	81.21%	83.13%
Current cigarette use			
Yes	5.30%	4.64%	6.04%
No	94.70%	93.96%	95.36%
Current alcohol drinking			
Yes	25.22%	24.03%	26.45%
No	74.78%	73.55%	75.97%
Sports team participation			
Yes	53.95%	51.97%	55.92%
No	46.05%	44.08%	48.03%
Got 8 or more hours of sleep			
Not meet	75.99%	75.14%	76.82%
Meet	24.01%	23.18%	24.86%
Spent 3 or more hours/day on ST			
Not meet	24.40%	23.59%	25.22%
Meet	75.60%	74.78%	76.41%
At least 60 min/day PA for all 7 days			
Not meet	75.42%	74.46%	76.35%
Meet	24.58%	23.65%	25.54%
Number of guidelines met			
0	12.27%	11.72%	12.83%
1	56.21%	55.41%	57.01%
2	26.58%	25.88%	27.29%
3	4.94%	4.57%	5.33%
Combination of guidelines met			
Meet none	12.27%	11.72%	12.83%
PA only	5.06%	4.72%	5.42%
ST only	46.43%	45.45%	47.40%
Sleep only	4.72%	4.41%	5.06%
PA + ST	12.24%	11.66%	12.84%
PA + Sleep	2.34%	2.12%	2.59%
ST + Sleep	12.00%	11.49%	12.54%
Meet all	4.94%	4.57%	5.33%

PA, physical activity; ST, screen time.

Table 3 explores the correlation between compliance with 24-hour movement guidelines and weight status. The results indicate a clear trend: lower adherence to movement guidelines correlates with worse weight status. Participants who not meeting any guidelines (OR = 1.38, CI = 1.20–1.58, *p* < 0.001), 1 guideline (OR = 1.42, CI = 1.28–1.58, *p* < 0.001), and 2 guidelines (OR = 1.18, CI = 1.20–1.58, *p* < 0.001) were more associated with worse weight status, compared with those who met the 3 guideline. Not meeting any guidelines is correlated with the highest odds of worse weight status, with statistically significant results. The odds ratios decrease as the number of guidelines being followed increases, suggesting a protective impact of increased PA, limited ST, and sufficient sleep. In terms of meeting specific behavioural guidelines, individuals who adhered to all the requirements were least likely to have an unfavourable weight status. Whereas participants who met only SB were most possible to have an unfavourable weight status (OR = 1.51, CI = 1.36–1.69, *p* < 0.001).

**Table 3:** Association between adherence to the 24-hour movement guidelines and weight status in overall sample.

Outcome: body weight	OR	95%CI		*p*
*Number of guidelines met (reference group = 3)*				
0	1.38	1.20	1.58	0.000
1	1.42	1.28	1.58	0.000
2	1.18	1.04	1.33	0.009
*Combinations of guidelines met (reference group = meet all)*				
Meet none	1.40	1.22	1.61	0.000
Meet PA only	1.02	0.86	1.20	0.833
Meet ST only	1.51	1.36	1.69	0.000
Meet Sleep	1.41	1.21	1.64	0.000
Meet PA + ST	1.10	0.96	1.27	0.173
Meet PA + Sleep	0.94	0.78	1.14	0.526
Meet ST + Sleep	1.34	1.18	1.52	0.0000

OR: odd ratio; CI: confidence interval; PA, physical activity, ST, screen time. All models controlled for sex, age, race/ethnicity, grade, eating habits, cigarette use, alcohol drinking, sports team participation, and year of data collection.

The examination in Table 4 stratifies the sample by sex and assesses the same associations as Table 3. The impact of not adhering to movement guidelines is more pronounced in boys than in girls. For boys who did not fulfill any of the criteria (OR = 1.63, CI = 1.37–1.93, *p* < 0.001), 1 guideline (OR = 1.49, CI = 1.31–1.70, *p* < 0.001), and 2 guidelines (OR = 1.16, CI = 1.00–1.34, *p* = 0.048) were associated with unfavourable weight status compared with who met all guidelines were more associated. In contrast, the effects for girls are less severe, with only certain combinations of guideline adherence showing significant associations.

**Table 4:** Association between adherence to the 24-hour movement guidelines and weight status in the sample by sex.

Outcome: body weight	OR	95%CI		*p*
Girl				
*Number of guidelines met (reference group = 3)*				
0	1.09	0.85	1.40	0.507
1	1.30	1.03	1.63	0.028
2	1.15	0.90	1.48	0.254
Boy				
*Number of guidelines met (reference group = 3)*				
0	1.63	1.37	1.93	0.000
1	1.49	1.31	1.70	0.000
2	1.16	1.00	1.34	0.048
Girl				
*Combinations of guidelines met (reference group = meet all)*				
Meet none	1.10	0.85	1.41	0.465
PA only	0.66	0.47	0.93	0.016
ST only	1.36	1.08	1.71	0.010
Sleep only	1.24	0.95	1.61	0.119
PA + ST	1.11	0.85	1.45	0.455
PA + Sleep	1.23	0.81	1.89	0.334
ST + Sleep	1.19	0.93	1.54	0.172
Boy				
*Combinations of guidelines met (reference group = meet all)*				
Meet none	1.65	1.39	1.96	0.000
PA only	1.17	0.96	1.43	0.123
ST only	1.59	1.39	1.82	0.000
Sleep only	1.50	1.22	1.83	0.000
PA + ST	1.08	0.92	1.28	0.335
PA + Sleep	0.86	0.68	1.10	0.241
ST + Sleep	1.41	1.18	1.68	0.000

OR: odd ratio; CI: confidence interval; PA: physical activity, ST: screen time. All models controlled for age, race/ethnicity, grade, eating habits, cigarette use, alcohol drinking, sports team participation, and year of data collection.

Table 5 categorizes results by age, showing that younger participants (ages 14 and 15) experience more pronounced negative outcomes from lower guideline adherence compared to older adolescents. Each age group demonstrates a trend where not meeting guidelines correlates with poorer weight status, with significant results particularly in the 14 and 15-year age groups. In the 14-year age group, participants who did not meet any guidelines (OR = 1.55, CI = 1.12–2.14, *p* = 0.008), 1 guideline (OR = 1.46, CI = 1.14–1.88, *p* = 0.003) were more correlated with unfavourable weight status compared to meeting all guidelines. In the 15-year age group, participants who did not meet any guidelines (OR = 1.44, CI = 1.10–1.88, *p* = 0.008), and 1 guideline (OR = 1.48, CI = 1.17–1.86, *p* = 0.001) were all more correlated with unfavourable weight status compared to meeting all guidelines.

**Table 5:** Association between adherence to the 24-hour movement guidelines and weight status in the sample by age.

Outcome: body weight	OR	95%CI		*p*
**14 years**				
Number of guidelines meet				
0	1.55	1.12	2.14	0.008
1	1.46	1.14	1.88	0.003
2	1.27	0.99	1.62	0.056
**15 years**				
Number of guidelines meet				
0	1.44	1.10	1.88	0.008
1	1.48	1.17	1.86	0.001
2	1.24	0.98	1.57	0.068
**16 years**				
Number of guidelines meet				
0	1.19	0.88	1.61	0.264
1	1.38	1.06	1.79	0.016
2	1.08	0.84	1.40	0.529
**17 years**				
Number of guidelines meet				
0	1.36	1.01	1.82	0.042
1	1.35	1.04	1.75	0.026
2	1.10	0.84	1.43	0.491
**14 years**				
Combination of guidelines meet				
Meet none	1.58	1.15	2.18	0.005
PA only	0.92	0.63	1.35	0.681
ST only	1.58	1.22	2.05	0.001
Sleep only	1.57	1.05	2.34	0.029
PA + ST	1.28	0.94	1.74	0.116
PA + Sleep	0.87	0.59	1.29	0.489
ST + Sleep	1.41	1.08	1.83	0.011
**15 years**				
Combination of guidelines meet				
Meet none	1.46	1.12	1.91	0.006
PA only	1.02	0.73	1.43	0.911
ST only	1.61	1.28	2.02	0.000
Sleep only	1.31	0.95	1.81	0.096
PA + ST	1.11	0.86	1.44	0.405
PA + Sleep	1.18	0.80	1.73	0.397
ST + Sleep	1.42	1.10	1.84	0.007
**16 years**				
Combination of guidelines meet				
Meet none	1.20	0.89	1.62	0.229
PA only	1.13	0.74	1.73	0.569
ST only	1.43	1.11	1.84	0.007
Sleep only	1.42	1.02	1.99	0.039
PA + ST	1.01	0.75	1.34	0.966
PA + Sleep	0.85	0.56	1.28	0.424
ST + Sleep	1.24	0.95	1.64	0.118
**17 years**				
Combination of guidelines meet				
Meet none	1.39	1.03	1.86	0.029
PA only	0.94	0.62	1.40	0.747
ST only	1.43	1.10	1.87	0.009
Sleep only	1.35	0.91	2.00	0.131
PA + ST	1.05	0.80	1.38	0.732
PA + Sleep	0.73	0.41	1.31	0.286
ST + Sleep	1.25	0.91	1.72	0.160

OR: odd ratio; CI: confidence interval; PA: physical activity, ST: screen time. All models controlled for sex, race/ethnicity, grade, eating habits, cigarette use, alcohol drinking, sports team participation, and year of data collection.

Table 5 presents the associations between guideline adherence and weight status across different racial and ethnic groups. White and Hispanic/Latino participants exhibit stronger negative outcomes from poor guideline adherence compared to other groups. White participants who did not adhere to any criteria (OR = 1.35, CI = 1.10–1.65, *p* = 0.004), and 1 guideline (OR = 1.37, CI = 1.16–1.62, *p* < 0.001) were all more correlated with unfavourable weight status compared to meeting all guidelines.

Hispanic/Latino participants who did not meet any guidelines (OR = 1.35, CI = 1.08–1.72, *p* = 0.011), 1 guideline (OR = 1.53, CI = 1.22–1.92, *p* < 0.001), and 2 guideline (OR = 1.24, CI = 1.01–1.53, *p* = 0.039) were all more correlated with unfavourable weight status compared to meeting all guidelines. For Black or African American participants, the associations are present but less consistently significant. This table underscores the varied impact of lifestyle behaviours across different racial and ethnic backgrounds.

## 4 Discussion

Based on nationally representative data from the YRBS combining different survey cycles (2017, 2019 and 2021 survey year), the goal of this research was to find the relationship between adhering to the 24-hour movement guidelines and body weight in teenagers aged 14–17. The key results of this study are as follows: (1) meeting fewer recommendations in the 24-hour movement guidelines is correlated with higher odds of overweight/obesity (OW/OB); (2) meeting PA guidelines only or the mixtures with PA guidelines is not significantly correlated with OW/OB; and (3) patterns in the connection between the quantity of suggestions in the 24-hour movement guidelines remain inconsistent across sex, age, and race/ethnicity subgroups.

### 4.1 Correlation between meeting the 24-hour movement guidelines and OW/OB

Numerous prior studies conducted in countries such as Australia, Canada, and China have shown that meeting fewer recommendations within the 24-hour movement guidelines is positively correlated with OW/OB, even after adjusting for variables such as dietary habits and weight loss efforts (27–29). Furthermore, two recent reviews indicate that following more suggestions within the 24-hour movement guidelines is linked to a degressive risk of OW/OB (1, 30). The findings of this study are consistent with these previous studies, indicating that insufficient adherence to the 24-hour movement guidelines is associated with an increased risk of OW/OB. Several mechanisms underpin these findings, highlighting that adequate PA coupled with decreased sedentary behaviour can lower energy intake, thereby potentially reducing OW/OB prevalence (31). Additionally, adherence to the 24-hour movement guidelines has been related to improved sleep patterns. A meta-analysis by Fatima et al. demonstrated that good quality of sleep correlates with a lower risk of OW/OB among young people (32). Insufficient sleep duration is also connected to a lower basal metabolic rate, reduced PA, and increased sedentary behaviour in children. Similarly, short sleep duration significantly heightens obesity risk in a dose–response manner due to endocrine and metabolic alterations and heightened appetite, which leads to more caloric intake, greater systemic inflammation, and lower PA due to daytime sleepiness (33). Healthy behaviours, including proper dietary patterns and adherence to the 24-hour movement guidelines, often cluster within the youth and teenagers, suggesting an interaction between movement recommendations and dietary intake and quality. Spending a lot of time in front of screens and not getting enough sleep have been linked to consuming more food., whereas regular PA can help regulate appetite. Thus, adhering to 24-hour movement guidelines might result in improved diet quality, an essential health-related behaviour, which subsequently lowers the risk of OW/OB. However, our results differ from some studies that found no association between fulfilling all three suggestions within the 24-hour movement guidelines and OW/OB. One possible reason for this discrepancy may be the very few compliances with all three recommendations in those studies. Despite some conflicting evidence, this study provides new insights and underscores the potential role of compliance with the 24-hour movement guidelines in preventing OW/OB in teenagers. Based on this analysis, it is essential to encourage good health 24-hour movement behaviours during adolescence, not only because they are cross-sectionally related to excess weight (as shown in this study, OW/OB) but also because they are prospectively linked to obesity in adulthood.

In some previous studies, such as those by Chen et al., Zhu et al., and Hui et al., adherence to PA guidelines and combinations thereof may not significantly associate with OW/OB in adolescents. The current research highlights the role of PA in adolescent weight management, supported by existing literature. This role can be attributed to PA’s impact on limiting excessive caloric intake and promoting healthy lifestyle behaviours (as noted earlier) (34, 35). This study aligns with existing literature indicating that if meeting PA guidelines and the combinations of PA or other guidelines, their associations with OW/OB are not significant in adolescents. These are responsible for healthy weight status maintenance. According to this finding, the importance of MVPA in health promotion and disease prevention (including OW/OB) has been emphasized again in adolescents. However, our results and current survey data both show low or not satisfied prevalence rate of sufficient MVPA (meeting the PA guidelines). Also, given that low prevalence rate of meeting the 24-hour movement guidelines is largely because of the insufficient PA, increasing adolescents’ PA participation is a priority. Future research should further explore the combined impact of PA, sleep, and sedentary behaviour on OW/OB using longitudinal designs and objective measures. Additionally, addressing the low adherence to these guidelines, particularly PA, should be a priority in future interventions to reduce adolescent obesity.

### 4.2 Correlation between meeting the 24-hour movement guidelines and OB/OW by age, sex, and race/ethnicity

Previous studies have shown demographic-related differences in the correlation between adherence to the 24-hour movement guidelines (including both the number and specific combinations of guidelines) and overweight/obesity (36, 37). In the current study, similar variations were observed by age, sex, and race/ethnicity, highlighting consistent findings with earlier research. In specific, the current study observed that in girls, results on the correlation between quantity of suggestions in the 24-hour movement guidelines were different from the those in boys (36). Similar patterns were also found in age subgroups. The different paths of teenager’s weight are due to age-and sex related difference in the process for growth. Regarding age-related variations in the associations between 24-hour movement guidelines and OW/OB, some studies support these findings. This may be due to a decline in parental belief impact (i.e., parental role model, motivation, guidelines, and limitations within the family) of social and environment factors on health behaviours as children grow into teenagers (38). Adolescents may experience fewer parental restrictions on lifestyle behaviours than children, thereby potentially leading to unhealthy weight status. One of the most extensively studied social factors in relation to the home environment is family socioeconomic status (SES), which includes factors such as parental education, family income, and more, as highlighted in a systematic review (39). Living in poverty has long been associated with negative health outcomes, impaired development in children, and poor health in adulthood (40). The differences across race/ethnicity were also observed. Previous studies have demonstrated the race-related differences in health behaviours, suggesting that White adolescents could have greater compliance to the 24-hour movement guidelines compared to their non-White counterparts (41). Likewise, previous research has reported that White adolescents prefer to participate in healthy patterns of movement behaviours when studied in isolation (e.g., sufficient levels of PA). Based on these results, we could imply that White adolescents may exhibit better health outcomes (42). Race/ethnicity-related health disparities in adolescents are a concerning public health problem in many countries. Based on the intersectionality framework, in racially/ethnically diverse countries, race/ethnicity is often times intertwined with other identities such as gender, income, class or immigrant status that make certain population groups more vulnerable in terms of access to resources and services such as knowledge that people can engage in healthy movement behaviours. Moreover, family structure is also an important factor within the social and environmental. Family structure plays a crucial role in child development, as it influences the caregiving environment, including the levels of parenting, economic resources available or invested in children, and the nature of their relationships with caregivers. When family structures change, the distribution of family resources, parental investments, and children’s caregiving environments are likely to change as well (43). On an international scale, institutional and political factors also play a significant role in shaping these dynamics. Previous studies have examined global strategies and institutions related to sports policy. PA has been positively associated with factors such as GDP, public health expenditures, media independence, political stability, government effectiveness, the rule of law, and control of corruption. In addition to these, several potential explanations can account for this inconsistency. First off, in precise assessments of movement behaviours may due to measurement errors that were collected form the self-reported questionnaires potentially skewing the connection between these behaviours and OB/OW. Secondly, evidence casts doubt on the independent connection between ST, SLP, and OW/OB among young populations, suggesting that ST and SLP may not be factors in OW/OB (11). Diet is a further explanation. Studies have indicated a connection between childhood and teenagers OW/OB and nutrition (44, 45). Although this study included eating-related variables, it did not collect data on some energy-related habits. Diet can lead to superfluous carol intake energy intake, lead to OW/OB. Thus, even if young individuals’ adherent to the 24-hour movement guidelines, those with unhealthy diets may still be more likely for OW/OB. These discoveries might inform sex-, age, and race/ethnicity-specific weight interventions and managements. Future research examining the interplay between movement guidelines and dietary habits could offer a more comprehensive approach to managing adolescent weight. These insights could inform targeted interventions aimed at reducing OW/OB disparities across diverse demographic groups.

### 4.3 Strengths and limitations

Several limitations must be acknowledged to better understand the findings. First, the reliance on self-reported measures for both the independent and dependent variables—such as adherence to the 24-hour movement guidelines, weight status, and covariates like age, sex, and race/ethnicity—introduces potential biases. Although the questionnaires used in the study are validated, self-reported data can be prone to inaccuracies, including recall bias, where participants may fail to accurately remember or report past behaviours, and social desirability bias, where respondents may provide answers, they believe are socially acceptable rather than their true behaviours. To address these issues, future studies could incorporate objective, device-based behavioural measures, such as accelerometers and pedometers, which would offer more accurate data on PA levels and movement behaviours.

Another significant limitation is the cross-sectional design of the study, which prevents the establishment of cause-and-effect relationships between variables. As a result, while the study identifies associations between adherence to movement guidelines and weight status, it is unclear whether adherence directly influences weight status or whether other unmeasured factors, such as genetics or environmental influences, contribute to these outcomes. This limitation highlights the need for longitudinal studies or experimental designs in future research to better establish causal links and account for the dynamic nature of health behaviours over time. Despite these limitations, a few strengths of the study are worth noting. These include the use of a large, nationally representative sample, which enhances the generalizability of the findings, and the consideration of eating-related factors, which are known to influence weight status but were often overlooked in previous studies. By addressing the limitations outlined and incorporating more robust methodologies, future research could provide a clearer and more comprehensive understanding of the factors influencing adolescent weight status.

## 5 Conclusion

This research suggests that meeting the 24-hour movement guidelines can significantly help in avoiding weight-related problems in American teenagers, with pronounced differences across sex, age, race/ethnicity subgroups. To validate these preliminary findings, future research should employ longitudinal designs to examine the differences among various age groups, sexes, and races, and to determine if promoting adherence to these movement guidelines effectively mitigates weight-related issues during adolescence.

## Data Availability

The original contributions presented in the study are included in the article/supplementary material, further inquiries can be directed to the corresponding author.
